# Reading performance is not affected by a prism induced increase of horizontal and vertical vergence demand

**DOI:** 10.3389/fnhum.2014.00431

**Published:** 2014-06-17

**Authors:** Muriel Dysli, Nicolas Vogel, Mathias Abegg

**Affiliations:** ^1^Department of Ophthalmology, Inselspital, University of BernBern, Switzerland; ^2^Graduate School for Cellular and Biomedical Sciences, University of BernBern, Switzerland

**Keywords:** dyslexia, reading, vergence, strabismus, phoria

## Abstract

**Purpose:** Dyslexia is the most common developmental reading disorder that affects language skills. Latent strabismus (heterophoria) has been suspected to be causally involved. Even though phoria correction in dyslexic children is commonly applied, the evidence in support of a benefit is poor. In order to provide experimental evidence on this issue, we simulated phoria in healthy readers by modifying the vergence tone required to maintain binocular alignment.

**Methods:** Vergence tone was altered with prisms that were placed in front of one eye in 16 healthy subjects to induce exophoria, esophoria, or vertical phoria. Subjects were to read one paragraph for each condition, from which reading speed was determined. Text comprehension was tested with a forced multiple choice test. Eye movements were recorded during reading and subsequently analyzed for saccadic amplitudes, saccades per 10 letters, percentage of regressive (backward) saccades, average fixation duration, first fixation duration on a word, and gaze duration.

**Results:** Acute change of horizontal and vertical vergence tone does neither significantly affect reading performance nor reading associated eye movements.

**Conclusion:** Prisms in healthy subjects fail to induce a significant change of reading performance. This finding is not compatible with a role of phoria in dyslexia. Our results contrast the proposal for correcting small angle heterophorias in dyslexic children.

## INTRODUCTION

Dyslexia is a developmental reading disorder that affects language skills. It is the most common neurobehavioral disorder in children with a prevalence between 5 and 20% of school-aged children in the United States (Handler and Fierson, 2011). It is characterized by difficulties with decoding, fluent word recognition, rapid automatic naming, and/or reading-comprehension skills (Handler and Fierson, 2011). By definition, congenital dyslexia is not caused by an organic deficit. The etiology is unclear and subject to an ongoing debate, see for example Olulade et al. (2013), Quercia et al. (2013).

It is known that eye movements during reading are abnormal in dyslexia: high numbers of regressive saccades and unstable fixation are the issues which are the most often mentioned (Pavlidis, 1981; De Luca et al., 1999; Trauzettel-Klosinski et al., 2010). Bucci et al. (2012) found that deficits are not limited to reading but also visual search performance is decreased in dyslexic children. Whether altered eye movements are the cause or rather the consequence of dyslexia is still debated (Livingstone et al., 1991; Skottun and Skoyles, 2006; Stoodley and Stein, 2013).

On the search for simple and reversible causes for their children’s learning deficits, many parents are receptive for alternative explanations. Especially latent strabismus (heterophoria) is commonly blamed to cause reading difficulties (Pestalozzi, 1992; Motsch and Muhlendyck, 2001; Abdi and Rydberg, 2005). In Germany, Austria, and Switzerland a large group of behavioral optometrists and opticians follow the school of Haase (1995) who aims at correcting small phoric angles in children and adults with various complaints including reading difficulties. Similar approaches are found in other countries. Accordingly, “visual therapies” and “visual trainings” aiming at improving the visual deficit are commercially offered with the explicit or implicit promise to improve dyslexia. Within the commuting area of the University Eye Clinic in Bern, where this study was conducted, there are at least six independent sites that offer visual training for reading problems. Based on these numbers, we estimate the prevalence of institutions offering visual therapies at 7.5 per 100’000 school-aged children. The associated socioeconomic costs are likely to be considerable. The techniques applied by visual therapists, their goal and the underlying theoretical framework are most heterogenous. Many share the concept however, that small uncorrected latent strabismus angles (phorias), in particular vertical phoria, lead to impaired reading. In consequence correction of small phoria angles is widespread. However, the efficacy of this has been questioned because of lack of positive evidence and on the basis of theoretical considerations (Gerling et al., 2000; Simonsz et al., 2001; Kromeier and Kommerell, 2006).

In the current study we approach the issue from a different viewpoint. We directly test the hypothesis that changes in vergence demands can cause reading deficits. For this we imposed increased vergence demands on healthy subjects and measured reading speed subsequently. This manipulation allows determining whether the vergence tone is or is not causally linked to reading efficacy. These experiments thus provide an independent line of evidence on the subject of vergence and dyslexia.

## MATERIALS AND METHODS

### SUBJECTS

Sixteen healthy subjects with a median age of 23.5 years (range 19–58 years) participated (nine females, seven males). 12 of the subjects were recruited among medical students. All were native Swiss German speakers, none of them self-reported to suffer from dyslexia. All participants had normal or corrected-to-normal vision and none of the subjects had a manifest strabismus or complained about double vision. 10 subjects were emmetropic, four subjects had mild or moderate myopia [<6 prism diopters (pdpt)], two subjects had high myopia (>6 pdpt). One subject was presbyopic. The high myopic subjects were recorded while wearing contact lenses, the other subjects were tested without optical correction. This was possible because the text size was chosen several orders of magnitudes above the lowest optical resolution of the tested subjects. Habitual phoria was tested with the alternating prism cover test. The study was conducted with approval of the local ethic committee Bern, Switzerland and all the subjects gave informed consent in accordance with the Declaration of Helsinki.

### EXPERIMENTAL SETUP

Experiments were conducted on a video-based eye-tracking system (EyeLink1000, SR Research). The subjects’ head was stabilized with a chin and a forehead rest. Stimuli were presented on a 19” CRT screen (View sonic Graphics Series G220fb) with a spatial resolution of 1024 × 768 pixels and a refresh rate of 85 Hertz (Hz). The screen was positioned 53.2 cm from the subjects forehead and spanned a visual angles of 41.2° horizontally and 31.5° vertically. Voices were recorded using a microphone that was attached to the chin rest; the voice recordings were synchronized with the onset of stimulus presentation. Eye movements of the left eye were recorded with an infrared camera at a sample rate of 2000 Hz.

### EXPERIMENTAL PROTOCOL

Each subject was instructed to read out loud 16 paragraphs of a German text. The paragraphs were not consecutive and arbitrarily selected from the book “Die Blendung” by Canetti (1980) to guarantee consistent text style. Subjects were instructed to read as rapidly and as accurate as possible as soon as the text appeared on the screen. The presentation of the text was manually stopped after the last word was spoken. After each paragraph, subjects were asked if they experienced double vision while reading. After each text, subjects were asked three multiple choice questions. The questions would refer to a detail of the preceding text requiring understanding of the text. An example is: “What has Kien forgiven his brother?” Along with the questions, three possible responses were provided and only one of which was correct. The subjects were to choose the best response by keypress response. For the example given above, the possible responses were: “(A) his beauty, (B) his weak character, (C) his change of specialization.”

Each paragraph consisted of 138 ± 3.6 words [mean ± standard deviation (SD)] or 861 ± 8.8 letters and 11 lines. Texts were presented with the font Times New Roman (font size 20 points, line space 1.5). The 16 paragraphs were alternatingly read with either normal view (serving as control) or with prisms. Prisms were placed directly in front of the right eye such that the monitor could only be viewed through the prisms. Convergence tone was increased by placing a prism base laterally, leading to an exophoric eye position, and decreased by placing it base medially, leading to an esophoric eye position, both conditions requiring compensation. For each of the two horizontal vergence conditions we tested 2, 4, and 6 prism diopters (pdpt; 1 pdpt *0.57 = 1°). Moreover, we tested two magnitudes of vertical placed prisms (1 and 2 pdpt). As a hyperphoria of one eye may be interpreted as hypophoria of the other eye, we only simulated hyperphoria of the right eye. During normal view (without prism) a plano glass was placed in front of the right eye. Prisms were always placed in front of the right eye right before the onset of the paragraph and were removed after the subjects had finished reading, i.e., after 2–4 min.

In order to counterbalance for possible learning and fatigue effects and to control for variations in difficulties of the paragraphs, we pseudo-randomized the allocation of the control texts and the texts read with prisms such that all texts were used once during the whole experiment. This order was changed for every subject, resulting in 16 sequences for 16 subjects.

### DATA ANALYSIS

To determine reading speed, we divided the duration from onset of text presentation until the last word was spoken by the number of letters per paragraph, resulting in number of read letters per second (s). To analyze attention and comprehension, we calculated a comprehension score for each subject for each prism condition. The comprehension score was defined as the ratio of correct answers and questions asked. For our experiment we thus divided the number of correct answers per condition by 3 (number of questions for each text). Thus guessing alone would result in a comprehension score of 33%.

For eye movement analysis, we determined the mean saccadic amplitude, the number of saccades required to read 10 letters, and the proportion of regressive saccades for each paragraph. To avoid microsaccades and saccades non-related to reading, we excluded all saccades <1° and >20° and all saccades in vertical direction. For each subject and each condition we determined the average duration of the performed fixations. For fixation detection the default parameters predefined by the eyetracker were used. As an additional measure of the fixation characteristics we determined the first fixation duration. For this we measured the duration of the first fixation on all 5–10 letter words that were located within the central one third of the text block. For the same words we determined the gaze duration, i.e., the sum of all firstpass fixations on a given word. Fixations resulting from regressive saccades are not included in this analysis. For all eye movement analysis we used SR Research EyeLink Data Viewer V 1.11.1 and Microsoft Excel.

For statistical analysis we used a linear mixed effects model with reading speed, comprehension score, fixation duration, saccade amplitude, saccade per 10 letters, percentage of regressive saccades, first fixation duration, and gaze duration as dependent variables. Prism condition was used as independent variable. Subjects and language materials were used as random effects. To select between different fitting models (random-intercept, random-slope, or combined) we used Akaike’s Information Criterion (AIC) and chose the best model by the principle “smaller-is-better.” *p*-values are reported. Analyses were performed using the MIXED procedure in SPSS (IBM SPSS Statistics 21).

## RESULTS

Thirteen of the subjects had no habitual phoria, the remaining three subjects had habitual phorias of 1 and 4 pdpt of exophoria, and 8 pdpt of esophoria respectively. Subjects with habitual phoria did not show different results compared to those without habitual phoria. None of the subjects self-reported reading difficulties and none complained about headache or task related eye pain during the experiment. Only one subject (without habitual phoria) mentioned transient double vision with 2 pdpt of vertical phoria.

**FIGURE 1:**
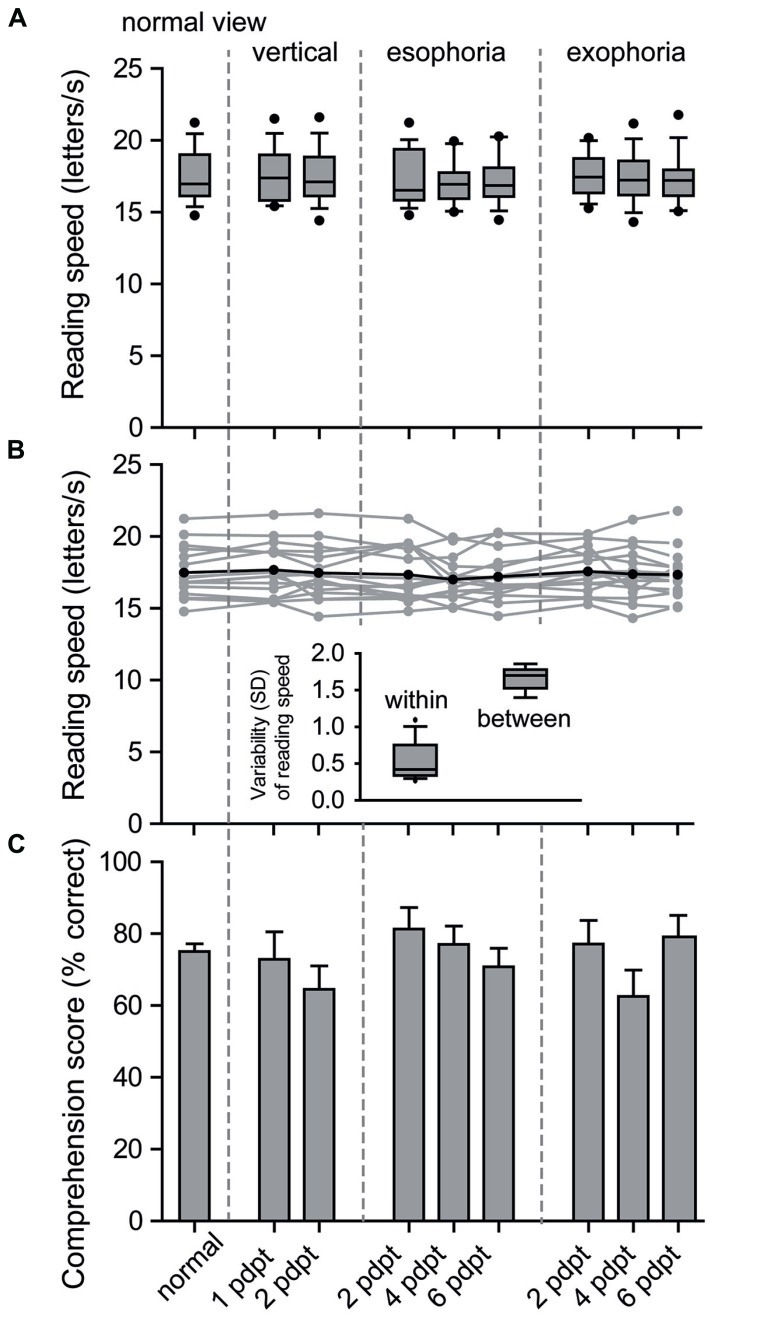
**Reading performance with prism induced changes of the vergence tone**. Decreased convergence tone, such as is experienced in esophoria, and increased convergence tone, as is experienced in exophoria was induced by placing prisms base medially and base laterally, respectively. Boxplots **(A)** show reading speed (letters/s) for normal view and each prism condition. The boxes contain the mean and 50% of the data, and the whiskers indicate 10/90% of the data. Mean reading speed of each subject reveals less intrasubject variability than between subject variability **(B**; gray = subjects, black = mean of all subjects). Inset figure shows mean variability of mean reading speed within subjects and between subjects. Bar chart showing the mean comprehension score (% correct answers) ± standard error of the mean for normal view and each prism condition **(C)**.

First, the effect of altered vergence tone on reading speed was investigated (**Table 1**, **Figure 1**). The linear mixed effects model with reading speed as dependent variable and prism conditions as independent variable showed no significant effect (*p* = 0.621; mean reading speed = 17.4 letters per second, range 14.3–21.8 letters per second).

**Table 1:** Mean reading speed, comprehension score, fixation duration, first fixation duration, and gaze duration (sum of all first pass fixations on a word) in milliseconds (ms) ± standard error of the mean for normal view and all prism conditions.

	Reading speed (letters/s)	Comprehension score (% correct)	Fixation duration (ms)	First fixation duration (ms)	Gaze duration (ms)
Normal view	17.49 ± 0.43	75.00 ± 2.12	212.73 ± 7.03	238.23 ± 9.29	281.36 ± 13.19
1 pdpt vertical	17.68 ± 0.44	72.92 ± 7.35	210.83 ± 6.40	230.83 ± 11.94	265.35 ± 17.78
2 pdpt vertical	17.46 ± 0.45	64.58 ± 6.23	211.55 ± 6.93	236.48 ± 11.35	287.42 ± 19.88
2 pdpt esophoria	17.34 ± 0.46	81.25 ± 5.87	212.95 ± 7.12	238.27 ± 12.03	281.74 ± 17.88
4 pdpt esophoria	17.02 ± 0.35	77.08 ± 4.86	212.39 ± 6.67	231.43 ± 11.84	288.98 ± 15.21
6 pdpt esophoria	17.19 ± 0.40	70.83 ± 5.00	216.44 ± 6.96	239.31 ± 17.08	275.41 ± 19.96
2 pdpt exophoria	17.57 ± 0.37	77.08 ± 6.40	214.19 ± 7.21	236.25 ± 11.84	264.16 ± 11.43
4 pdpt exophoria	17.38 ± 0.43	62.50 ± 7.14	215.52 ± 7.68	242.16 ± 14.46	286.22 ± 18.23
6 pdpt exophoria	17.35 ± 0.40	79.17 ± 5.80	218.45 ± 7.26	239.16 ± 16.06	309.97 ± 23.50

As vertical phoria is a common suspect for causing reading difficulties (Simons and Gassler, 1988), reading speed with vertically placed prisms was compared with normal viewing conditions with *a priori* paired *t*-tests. Both, normal viewing conditions compared with 1 pdpt (*t* = 1.6, *p* = 0.13) and with 2 pdpt (*t* = 0.2, *p* = 0.87) of vertical orientated prisms did not show significant effects on reading speed. Moreover, variability between the different prism conditions was significantly lower within subjects than between subjects (inset **Figure 1**; mean variability within subjects = 0.56 ± 0.06 [mean ± standard error of the mean (SEM)], variability between subjects = 1.66 ± 0.05 (mean ± SEM); *p* = < 0.0001, paired *t*-test).

Analyzing the comprehension score, we found no significant effect of prism conditions (*p* = 0.354). *A priori* contrast of normal viewing conditions with vertically placed prisms did not show any significant differences: neither for 1 pdpt (*t* = 0.3, *p* = 0.79) nor for 2 pdpt (*t* = 1.8, *p* = 0.09).

**FIGURE 2:**
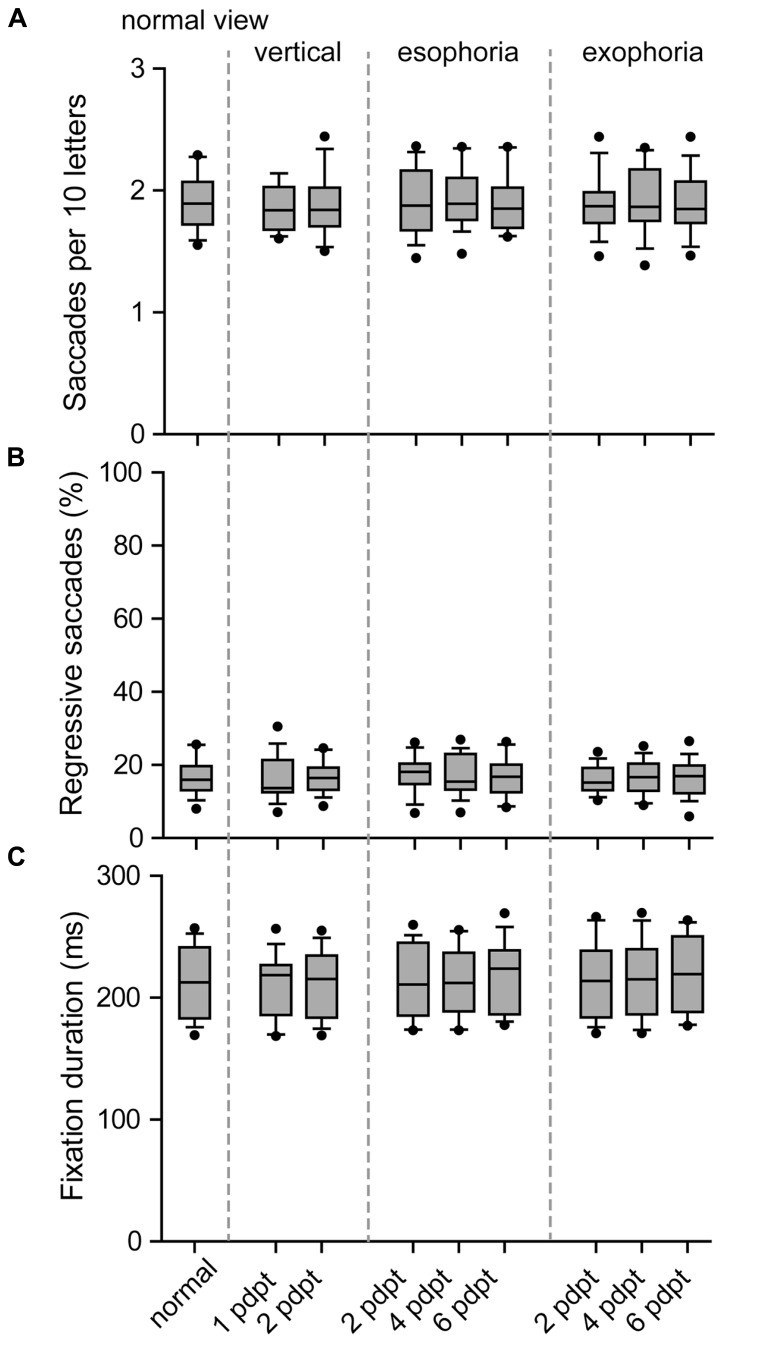
**Eye movements during reading with prisms**. Boxplots for number of saccades per 10 letters **(A)**, proportion of regressive saccades **(B)**, and average fixation duration **(C)**. The boxes contain the mean and 50% of the data, and the whiskers indicate 10/90% of the data.

The analysis of the eye movements that were made during reading with altered vergence tone failed to show a significant effect of prisms. Neither saccadic amplitudes (*p* = 0.718), saccades per 10 letters (*p* = 0.629), percentage of regressive saccades (*p* = 0.813; **Table 2**, **Figure 2**), fixation duration (*p* = 0.594; **Table 1**, **Figure 1**), first fixation duration (*p* = 0.993), nor gaze duration (*p* = 0.677) were significantly influenced by the prism conditions.

**Table 2:** Mean saccade amplitude, saccades per 10 letters and percentage of regressive saccades ± standard error of the mean for normal view and all prism conditions.

	Saccade amplitude (°)	Saccade per 10 letters	Regressive saccades (%)
Normal view	2.49 ± 0.08	1.90 ± 0.06	16.66 ± 1.18
1 pdpt vertical	2.51 ± 0.08	1.86 ± 0.05	16.29 ± 1.48
2 pdpt vertical	2.52 ± 0.08	1.88 ± 0.06	16.82 ± 1.10
2 pdpt esophoria	2.50 ± 0.08	1.91 ± 0.06	17.46 ± 1.23
4 pdpt esophoria	2.52 ± 0.09	1.94 ± 0.06	16.87 ± 1.32
6 pdpt esophoria	2.54 ± 0.10	1.90 ± 0.06	16.70 ± 1.32
2 pdpt exophoria	2.43 ± 0.06	1.89 ± 0.06	16.11 ± 0.89
4 pdpt exophoria	2.50 ± 0.07	1.91 ± 0.07	16.59 ± 1.15
6 pdpt exophoria	2.49 ± 0.08	1.89 ± 0.06	16.32 ± 1.22

## DISCUSSION

We found that acute prism induced horizontally and vertically altered vergence tone does not significantly affect neither reading speed, comprehension of content, nor reading associated eye movements. The low within subject variability of reading speed as compared to intersubject variability indicates that even a larger sample size would not significantly change our findings. This provides an independent line of evidence against the role of small phoria correction in reading disorder: if an acute change from the physiological state in a healthy subject does not impair reading, we presume that the inverse, a correction of a pathological phoria back to physiological orthophoria will not be useful to treat reading difficulties. The latter is in accordance with Wahlberg-Ramsay et al. (2012), who evaluated the binocular function (visual acuity, refractive error, best corrected visual acuity at distance and near, near point of convergence, amplitude of accommodation, stereopsis, phorias, fusional reserves) in dyslexic children in comparison to age-matched control children. They found indeed a reduced amplitude of accommodation in dyslexic children. But apart from this, there seems to be no difference between dyslexic and control children in terms of binocular function. They concluded that binocular deficits in dyslexic children are a result of the phonological deficit of dyslexia and not an underlying cause for dyslexia (Wahlberg-Ramsay et al., 2012). This finding is supported by Handler and Fierson (2011) who found no causal relation between reading ability and the binocular and accommodative status of randomly chosen children. Latvala et al. (1994) as well reported no ophthalmologic differences between dyslexic and control children regarding the visual acuity, cycloplegic refraction, the amount of phorias and tropias, stereo acuity, fusion, or accommodation. Only the convergence near point was more frequent ≥8 cm in the dyslexic group (Latvala et al., 1994). Even manifest strabismus (tropia) has not been associated with dyslexia (Cassin, 1975; Metzger and Werner, 1984).

Prism induced changes of phoria, as used in this experiment, are a model for habitual phoria with some shortcomings. First, we have tested young adults in our experiments while dyslexia is considered a problem of childhood. Furthermore, while phoric subjects experience a constant increased vergence demand in order to maintain binocular alignment, prisms may only cause acute and transient changes. The phoria simulated with prisms is thus in an unadapted state. This would rather lead to an overestimation of prism induced effects and thus is unlikely to explain our findings. Another problem with our model is that acute prism induced phoria may be compensated over time to result in orthophoria again. This effect is well known as “prism adaptation,” “vergence adaptation,” or “phoria adaptation” (Toole and Fogt, 2007). As we have not measured the actual phoria during the experiment, we cannot conclude that we truly measured reading with phoria. Possibly some phoria adaptation took place during our measurements. This is the reason for using the term “prism induced increase of vergence demand” rather than “phoria.” The increased vergence demand however, is a relevant aspect of phoric subjects. The fact that phoric patients have an increased vergence demand is obvious in patients with congenital fourth nerve palsy. Those patients are subject to a lifetime of increased vergence demand requiring compensation. As a consequence, vertical fusional range in those subjects is increased as compared to normal subjects (Kono et al., 2002). Nevertheless those patients are not known to have more difficulties with reading which is compatible with our results. The time course of phoria adaptation as measured by others suggests that phoria may be adapted over a time course of 60 min (Graf et al., 2003; Brautaset and Jennings, 2005). Since reading of a text block was achieved within 50 ± 0.3 s (mean ± SEM) we argue that reading was indeed done not only with increased vergence demand but with an actual phoria, which might have changed over time.

In our present study, fixation duration was not altered by reading with acute prism induced horizontally and vertically altered vergence tone. However, studies with strabismic children (Lions et al., 2013) and adult strabismic amblyopes (Kanonidou et al., 2010) have shown that those patients exhibit longer fixation durations. Concerning this aspect, we guess that the induced latent strabismus in our normal subjects might have a slightly different effect than in children with latent strabismus who are learning to read. It is known that patients suffering vergence insufficiency benefit from orthoptic exercises (Helveston, 2005; Scheiman and Beck, 2008), and there is some evidence that vergence training may improve eye movements during reading in children with strabismus (Gaertner et al., 2013). However, children with dyslexia are not necessarily strabismic.

In summary, our findings allow the conclusion that increased vergence demand, be it horizontally or vertically, does not affect reading performance or eye movements. Since vergence demand is increased in phoric subjects, our findings are not compatible with vergence demand as cause for reading difficulties. This corresponds well to our clinical observation that patients with large phorias may complain about double vision, eye strain, or headaches but not reading difficulties.

Our results are compatible with the finding that children and adults with dyslexia usually have normal binocular function (Handler and Fierson, 2011) and that children with significant eye movement and stability disorders (including nystagmus and strabismus) are no more likely to have dyslexia than children in the general population (Granet, 2011). This absence of evidence contrasts the recommendation for an ophthalmic workup in children with learning disabilities proposed by several groups (Pestalozzi, 1992; Motsch and Muhlendyck, 2001; Lack, 2010). Motsch and Muhlendyck (2001), for example evaluated the data of dyslexic children and found ocular disturbances in 28 of 33 children (mostly accommodative problems: uncorrected hyperopia, hypoaccommodation and/or exophoria compensated by accommodative convergence (pathophoria)), 26 showed improved reading after therapy. They therefore underline the importance of the correction of even small refraction and/or motility errors in the presence of reading and writing difficulties (Motsch and Muhlendyck, 2001). Similarly, Pestalozzi (1992) evaluated 281 dyslexics which then were corrected by Haase’s method of prismatic binocular full correction. Visual acuity as well as sensory adaptation improved. In 71%, the influence on dyslexia was good to very good. 17% showed no influence on dyslexia but got rid of asthenopic symptoms. Only 12% failed. The author’s point of view is that prismatic corrections may save energy as the patients have no longer to compensate their heterophoria themselves and thereby improve reading performance (Pestalozzi, 1992).

The main drawback of the above mentioned studies, which suggest a role of phoria in reading and learning deficits, is the lack of good control subjects. We suspect that the apparent benefits of prism correction could be explained by a placebo effect as well. However, our results are not a final proof for the absence of contribution of phoria on reading abilities in dyslexics. But given the lack of positive evidence for the benefit of phoria correction in dyslexic children without manifest strabismus and our result of no reading difficulties with acutely altered vergence tone, there is in our opinion strong evidence for the lack of benefit of phoria correction in dyslexic children. We suggest that those advocating prism correction to treat dyslexia are now challenged to provide positive evidence. This could be easily done with a randomized, double blind trial comparing children with prism glasses to children wearing placebo prisms.

## Conflict of Interest Statement

The authors declare that the research was conducted in the absence of any commercial or financial relationships that could be construed as a potential conflict of interest.

## AUTHOR CONTRIBUTIONS

Muriel Dysli: Analysis and interpretation of data, manuscript writing; Nicolas Vogel: Collection and analysis of data, proofreading of manuscript; Mathias Abegg: Conception and design of the experiments, analysis of data, manuscript writing.
